# Key Components of PPEO in Antagonizing Cerebral Ischemic Reperfusion Injury in Rats by Regulating Ferroptosis Through Arachidonic Acid Metabolic Pathway

**DOI:** 10.3390/cimb47110912

**Published:** 2025-11-03

**Authors:** Zilong Du, Fan Huang, Yilin Liang, Lu Xie, Wanxiang Hu

**Affiliations:** Department of Physiology, Pre-Clinical Science, Guangxi Medical University, Nanning 530021, China; 202320008@sr.gxmu.edu.cn (Z.D.); huangfan0816@sr.gxmu.edu.cn (F.H.); 202420059@sr.gxmu.edu.cn (Y.L.)

**Keywords:** cerebral ischemia–reperfusion injury, arachidonic acid metabolism, ferroptosis, PPEO, nootkatone, ACSL4-LPCAT3-ALOX15 axis

## Abstract

Cerebral ischemic reperfusion injury (CIRI) induces irreversible neurological dysfunction with high morbidity and mortality, yet effective clinical interventions remain limited. This study focused on ferroptosis in CIRI and explored the neuroprotective components and mechanisms of Pomelo peel essential oil (PPEO)—a product derived from Guangxi’s characteristic Shatian pomelo. Sprague-Dawley rats were used to establish two CIRI models: focal CIRI via Middle Cerebral Artery Occlusion (MCAO) and global CIRI via Cardiac Arrest/Cardiopulmonary Resuscitation (CA/CPR). Analyses were conducted using metabolomics, transcriptomics, histopathological staining, biochemical assays, RT-qPCR, Western blotting (WB), and molecular docking. Metabolomic results showed altered lipid-related metabolites in both models, predominantly unsaturated fatty acids and components of the arachidonic acid (AA) metabolic pathway. Transcriptomic analysis revealed significant upregulation of *PTGS1/2* in the MCAO model. Nootkatone and β-pinene improved neuronal morphology, increased glutathione peroxidase 4 (GPX4) levels, and enhanced neurological scores. Notably, Nootkatone exhibited strong binding affinity to ALOX15, and reduced lipid metabolic disturbances in the CA/CPR model. AA metabolism varies with CIRI severity: it is inflammation-driven in focal CIRI and ferroptosis-associated in global CIRI. As a key component of PPEO, Nootkatone antagonizes ferroptosis via the ACSL4-LPCAT3-ALOX15 axis, offering a novel therapeutic target for global CIRI after CA/CPR.

## 1. Introduction

Clinically, CIRI occurs after thrombolytic treatment for ischemic stroke or following cardiac arrest/cardiopulmonary resuscitation (CA/CPR) [1]. Based on transient ischemic–hypoxic brain damage due to sudden interruption of cerebral blood flow, more serious secondary damage to brain tissue occurs when blood flow is restored again. This can result in a range of neurological deficits, including sensory disturbances, motor dysfunction, cognitive decline, and speech impairments, which may ultimately lead to severe disability or death [2]. Currently, a pharmacological strategy specifically designed to improve neurological recovery following CIRI is lacking. So it is impossible to overemphasize the necessity for ongoing research to elucidate the pathophysiological mechanisms underlying this condition. The identification of potential intervention targets and novel therapeutic strategies remains critical for the advancement of clinical management and improving the prognosis of the patient.

Recently, the neuroprotective potential of Pomelo peel essential oil (PPEO) has garnered growing interest, driven by the pursuit of developing locally sourced, food-derived therapeutic candidates [3,4,5]. PPEO is extracted from Shatian Pomelo peel, a specialty of Rong country, Guangxi. It belongs to the Citrus peel essential oil, which has significant antioxidant, antimicrobial, tumor inhibitory, and immunomodulatory effects due to the presence of terpenoids, phenols, and flavonoids [6,7]. Our previous study demonstrated that PPEO enhanced antioxidative capacity by upregulating the expression of SLC7A11 and GPX4 in vivo and in vitro, suggesting that its cerebroprotective effects against CIRI are mediated through suppression of ferroptosis via the classical redox pathway [8]. However, the specific bioactive constituents of PPEO and their precise mechanisms require further investigation.

Among the complex pathophysiological mechanisms involved in the development of CIRI, dysfunctional energy metabolism and oxidative stress damage play a key role in exacerbating the CIRI process [9]. Our team’s earlier research found that extracellular signal-regulated kinase (ERK) inhibitors [10], green tea polyphenols [11], and serum potassium [12] exert neuroprotective effects by alleviating lipid peroxidation accumulation and increasing ATP levels after CIRI in the MCAO or CA/CPR rat models. These findings indicate that pathological damage after CIRI revolves around metabolic and energy metabolic disorders. In recent years, biosequencing technologies have advanced rapidly, and multi-omics approaches have been widely employed to identify novel biomarkers and differentially expressed genes. It provides relatively precise exploration directions for disease research. A comprehensive and systematic study of metabolomic changes in different degrees of CIRI can provide a biomics basis for early diagnosis, improved prognosis, and therapeutic strategies for CIRI.

In the present study, we established two distinct models of CIRI-MCAO and CA/CPR- and applied integrated serum untargeted metabolomics and brain tissue transcriptomics to characterize the differential molecular alterations associated with varing severities of CIRI. At the same time, the key neuroprotective components of PPEO were identified. Subsequently, we explored the specific mechanisms of PPEO key components, tried to establish a credible laboratory basis for developing local neuroprotective drugs, and provided new strategies and ideas for clinical CIRI treatment.

## 2. Materials and Methods

### 2.1. Main Reagents

Carvacrol (CAR), Nootkatone (NK), and β-pinene (β-PIN) were purchased from Lemeitian Pharmaceutical. Tissue iron kit (Jiancheng, Nanjing, China, #A039-2-1), Malondialdehyde (MDA) kit (Jiancheng, Nanjing, China, #A003-1-2), Rat arachidonic acid (ARA) ELISA research kit (Meimian, Yancheng, China, #MM-0929R1), DAB chromogenic kit (Servicebio, Wuhan, China, #G1212-200T), RevertAidTM premixed reverse transcription kit (Thermo Fisher Scientific, Waltham, MA, USA, #M16325), SYBR green (Thermo Fisher Scientific, Waltham, MA, USA, #A25742), BCA kit (Beyotime Biotechnology, Beijing, China), Polyvinylidene fluoride (PVDF) membrane (Bio-Rad, Hercules, CA, USA), GPX4 antibody (Selleck, Houston, TX, USA, #F1580), GAPDH antibody (Servicebio, Wuhan, China, #P16858), ALOX15 antibody (Bioss, Beijing, China, #bs-6505R), LPCAT3 antibody (Abmart, Shanghai, China, #PK53132S), COX-2 antibody (Abmart, Shanghai, China, #TP5318F), ACSL4 antibody (Selleck, Houston, TX, USA, #F1153), Goat anti-rabbit recombinant secondary antibody (Proteintech, Wuhan, China, #RGAR006), Goat anti-rabbit recombinant secondary antibody (Zen-bio, Chengdu, China, #550037).

### 2.2. Extraction of PPEO Solution

Shatian pomeloes purchased from Rongxian, Guangxi, were washed with ultra-pure water and peeled after drying. Fresh peel (120 g) was cut into small pieces and placed into a 500 mL round-bottom flask. Ultra-pure water was added to bring the volume up to 400 mL, followed by the addition of 3.6 g NaCl (5%). Distillation was performed according to the method established by our research team in the previous [8]. The upper oil layer was carefully isolated when it reached a thickness of 3 mm. Then, it was kept sealed at 4 °C and stored in the dark for further use.

### 2.3. Animal Models and Experimental Grouping

Adult male Sprague-Dawley rats weighing between 180 and 220 g were purchased from the Guangxi Medical University Laboratory Animal Center in Nanning, China. The rats were fed standard chow and given free access to water for 3 d prior to the experiments. All animal protocols were conducted in accordance with ethical guidelines and approved by the Guangxi Medical University Animal Ethics Committee on 20 September 2023.

The MCAO model was established using the modified Longa method [13], in which the middle cerebral artery was occluded for 2 h and then reperfused. CA/CPR model was constructed in accordance with the protocol established by our research group in the previous study. We used transesophageal electrical stimulation to induce cardiac arrest and performed hands-only cardiopulmonary resuscitation 7 min later [14].

#### 2.3.1. Metabolomics and Transcriptomics Sequencing

Twelve male Sprague-Dawley rats were randomly divided into three groups (*n* = 4): the normal group (N), the MCAO group (MM), and the CA/CPR group (CM). 24 h after modeling, all rats were euthanized. Blood was collected from the abdominal aorta. The blood samples were left standing at 4 °C for 2 h, then centrifuged at 3000 rpm at 4 °C for 10 min. The supernatant was stored at −80 °C for metabolomics detection. Then, after decapitation, the surface blood of the brain tissue was washed with ice normal saline. The ischemic penumbra tissue was taken after removing the meninges, and stored at −80 °C for transcriptome sequencing.

#### 2.3.2. Preliminary Efficacy Exploration of Key Components of PPEO

Seventy-two male Sprague-Dawley rats were randomly divided into 12 groups (*n* = 3) in the two models, respectively. MCAO model: sham operation group (SHAM group), MCAO group, 10% DMSO (DMSO group), CAR 10 mg/kg (CAR10 group), CAR 20 mg/kg (CAR20 group), CAR 40 mg/kg (CAR40 group), NK 1 mg/kg (NK1 group), NK 5 mg/kg (NK5 group), NK 10 mg/kg (NK10 group), β-PIN10 mg/kg (β-PIN10 group), β-PIN20 mg/kg (β-PIN20 group) and β-PIN 40 mg/kg (β-PIN40 group). CA/CPR model: also divided into SHAM group, CA group, DMSO group, CAR10 group, CAR20 group, CAR40 group, NK1 group, NK5 group, NK10 group, β-PIN10 group, β-PIN20 group and β-PIN40 group. The modeling and sampling methods were the same as above. All drugs used normal saline containing 10% DMSO as solvent. After thread removal and reperfusion, except for the SHAM group, MCAO group, and CA group which were intraperitoneally injected with 0.9% normal saline, the other groups were injected with corresponding concentrations of drugs. The setting of drug concentration gradients was based on pre-experiments, combined with previous literature reports where these concentrations have been previously validated as non-harmful to health and the relative content of each component in PPEO [15,16,17,18,19,20].

#### 2.3.3. Study on the Mechanism of PPEO and Its Key Components Against CIRI

A total of 45 male Sprague-Dawley rats were randomly divided into 5 groups (*n* = 9): sham operation group (SHAM), CA group, 10% DMSO group (DMSO), NK 5 mg/kg group (NK), and PPEO 40 mg/kg group (PPEO). The modeling and sampling methods were the same as before. Ischemia time was extended to 8 min, and drugs were administered via femoral vein injection using a micro pump after the return of spontaneous circulation.

### 2.4. Metabolome Sequencing

The metabolites were detected using ultra-high-performance liquid chromatography-quadrupole time of flight-mass spectrometry (UPLC-QTOF-MS). Sample preparation followed previously reported methods, with slight modifications [21]. The analysis was performed on a Waters ACQUITY UPLC I-Class PLUS System (Waters, Milford, MA, USA), coupled with a SELECT SERIES Cyclic IMS (Milford, MA, USA) mass spectrometer and equipped with a Waters Acquity UPLC HSS T3 column (1.8 μm, 2.1 × 100 mm). The raw data from metabolomics were processed using Progenesis QI 2.0 (Waters, USA) for baseline filtering, peak identification, integration, retention time correction, and peak alignment. Retention times, mass-to-charge ratios, and peak intensities were recorded. The Human Metabolome Database (HMDB) was used to identify differential metabolites. Multivariate statistical analysis, including Principal Component Analysis (PCA) and Orthogonal Projections to Latent Structures Discriminant Analysis (OPLS-DA), was performed using SIMCA 14.1 (Umetrics, UMEA, Umeå, Sweden), with 200 permutation tests. Differential metabolites were selected using the criteria: score > 42 (out of 60), fold change >1.5 or <0.67, and *p* < 0.05, VIP > 1. The MetaboAnalyst 6.0 software (https://www.metaboanalyst.ca/ (accessed on 4 May 2024)) was used for ROC analysis and Kyoto Encyclopedia of Genes and Genomes (KEGG) pathway enrichment analysis. A clustering heatmap was also generated using an online tool (https://www.bioinformatics.com.cn/ (accessed on 4 May 2024)).

### 2.5. Transcriptome Sequencing

Total RNA was extracted from rat brain tissue using Trizol lysis. Transcriptomic sequencing was performed by Shanghai Sangon Company, using the Illumina HiSeq platform for high-throughput sequencing. The cDNA library was constructed using the Hieff NGS™ magnetic bead purification product, followed by PCR amplification. After quality assessment with FastQC (0.11.2), data were filtered using Trimmomatic. HISAT2 was used for efficient alignment of the quality-controlled data to the reference genome. RSeQC was used to verify alignment results. Gene expression was normalized using StringTie v2.2.3 with the TPM (transcripts per million) method. Differentially expressed genes (DEGs) were screened using DESeq with the threshold of q-value < 0.05 and fold change >2. DEGs were categorized based on log2 fold change into upregulated or downregulated genes. Gene Ontology (GO) analysis and KEGG pathway enrichment analysis were conducted using an online tool (https://www.bioinformatics.com.cn/ (accessed on 4 May 2024)).

### 2.6. Histopathological Staining

Fresh brain tissue samples were fixed for 24 h. Then, they underwent routine dehydration, xylene transparency, paraffin embedding, sectioning, and subsequent Hematoxylin and Eosin (H&E) staining, Nissl staining, and immunohistochemistry. The immunohistochemistry conditions were as follows: Antigen retrieval was performed using EDTA8.0 at 90 °C for 30 min. The membrane was blocked with 1% defatted milk adn incubated with the primary antibody (LPCAT3, Abmart, PK53132S, 1:200) overnight at 4 °C. Then it was incubated with the secondary antibody (S-vision, goat anti-rabbit) for 1 h at room temperature. Color development was achieved using DAB for 10 min, followed by washing with deionized water for 30 s. Hematoxylin staining for 1 min, and rinsing with deionized water.

### 2.7. Detection of Biochemical Indicators

Brain cortex tissue was collected 24 h after reperfusion. Tissue iron and MDA levels were measured using the Tissue Iron Reagent Kit and MDA Reagent Kit according to the manufacturer’s instructions.

### 2.8. Enzyme-Linked Immunosorbent Assay (ELISA)

Blood serum samples were collected from each group of rats 24 h post-reperfusion. Arachidonic acid levels in serum were quantified using the Arachidonic Acid ELISA Kit (Meimian, MM-0929R1) according to the manufacturer’s instructions.

### 2.9. Real-Time Fluorescent Quantitative PCR (RT-qPCR)

Total RNA was extracted from tissue and cells using chloroform and Trizol (1:5). Reverse transcription was performed using RevertAid™ Premixed Reverse Transcriptase Kit. The mRNA expression levels were detected using SYBR Green, and the qPCR reaction was carried out on a StepOnePlus™ Real-Time PCR System (Thermo Fisher Scientific, Waltham, MA, USA). *GAPDH*:(Sangon, B661204-0001) was used as the reference primer. The primer sequences for the genes are as follows (Table 1).

### 2.10. Molecular Docking

The active components’ 3D structures in SDF format were downloaded from PubChem, and the structures were converted into PDB format using OpenBabel 2.4.1 software. 3D crystal structures of target proteins were downloaded from the PDB database. Ligands and target proteins were converted to “pdbqt” format using AutoDockTools 1.5.7. Molecular docking was performed using AutoDock VINA 1.2.5, and the binding energies were calculated. The docking results were visualized using PyMOL 3.0.

### 2.11. Neurological Function Scoring

Neurological dysfunction in the MCAO rat model was assessed using the Zea Longa five-point scoring system [13], where 0 indicates no neurological deficit, 1 indicates a failure to extend the contralateral forelimb upon tail suspension, 2 indicates circling behavior, 3 indicates tilting toward the contralateral side, and 4 indicates no spontaneous movement or consciousness. For the CA/CPR model, the NDS scoring system was used [8].

### 2.12. Western Blotting

Protein lysates were prepared from brain tissues using RIPA lysis buffer containing protease and phosphatase inhibitors. Protein concentrations were measured using the BCA assay. Proteins were separated on 12% and 10% SDS-PAGE gels and transferred onto PVDF membranes. The membranes were blocked with 5% non-fat dry milk in TBS with 0.1% Tween-20 for 2 h at room temperature, and then incubated overnight with primary antibodies at 4 °C: GPX4 (1:1000), ALOX15 (1:1000), GAPDH (1:5000), LPCAT3 (1:1000), and ACSL4 (1:1000). The membranes were washed three times with TBST and incubated with horseradish peroxidase-conjugated secondary antibodies for 1 h. The signals were visualized using the Odyssey infrared imaging system (LI-COR, Lincoln, NE, USA) and analyzed using Image-J software (v1.33, NIH, Bethesda, MD, USA).

### 2.13. Cellular Thermal Shift Assay (CETSA)

Brain tissue samples were homogenized in RIPA lysis buffer containing protease and phosphatase inhibitors. The homogenate was then centrifuged at 12,000× *g* for 15 min at 4 °C to separate soluble proteins in the supernatant from tissue debris. The supernatants were incubated with either 20 μM Nootkatone or 0.1% DMSO for 1 h. Subsequently, the samples were centrifuged again at 12,000× *g* for 15 min at 4 °C. The supernatants were then divided into 8 aliquots, and each aliquot was heated for 10 min at different temperatures (47, 50, 53, 56, 59, 62, 65 and 68 °C) using a PCR machine. After heating, the samples were centrifuged at 16,000× *g* for 20 min at 4 °C. The supernatants were collected, mixed with loading buffer, boiled, and then subjected to Western blot analysis to assess the relative protein levels.

### 2.14. Statistical Analysis

Data were analyzed using GraphPad Prism 9.0.0. Results are presented as means ± standard deviation (SD). Differences between two groups were evaluated using Student’s *t*-test, and multiple comparisons were performed using one-way analysis of variance (ANOVA). SPSS 22.0 software (IBM Corporation, Armonk, NY, USA) was used for statistical analysis, with *p* < 0.05 considered statistically significant.

## 3. Results

### 3.1. Preliminary Screening of Key Components of PPEO

**Figure 1:**
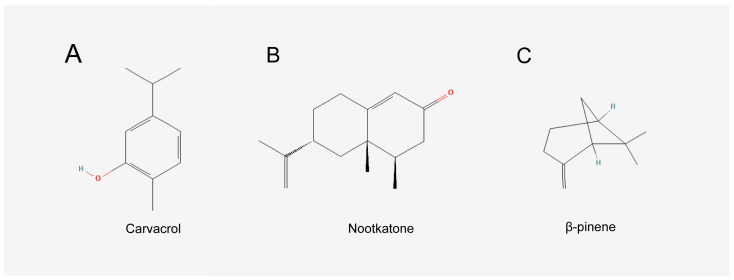
Chemical structure diagrams of the three components. (**A**) Chemical structure diagrams of Carvacrol. (**B**) Chemical structure diagrams of Nootkatone. (**C**) Chemical structure diagrams of β-pinene.

This study aims to further investigate and identify the specific effective components of PPEO that regulate cerebral ischemia–reperfusion injury (CIRI) and exert neuroprotective effects. Notably, previous studies have employed liquid chromatography-mass spectrometry (LC-MS) and network pharmacology analysis to screen potential bioactive components in PPEO. Though Limonene (50.67%) is the most abundant component in PPEO, it did not yield favorable results in our preliminary experiments and was therefore not included in subsequent analyses. Instead, three components—Carvacrol (CAR), Nootkatone (NK), and β-Pinene (β-PIN)—were identified as promising candidates (Figure 1). According to prior data, the relative concentrations of these three components in PPEO are 4.29% (CAR), 2.82% (NK), and 2.57% (β-PIN), with only minor variations across different batches [3,4,5]. Accordingly, these three components were selected to test their neuroprotective effects in both the middle cerebral artery occlusion (MCAO) and cardiac arrest/cardiopulmonary resuscitation (CA/CPR) models.

#### 3.1.1. Both Nootkatone and β-Pinene Exert Good Neuroprotective Effects in Both Models

**Figure 2:**
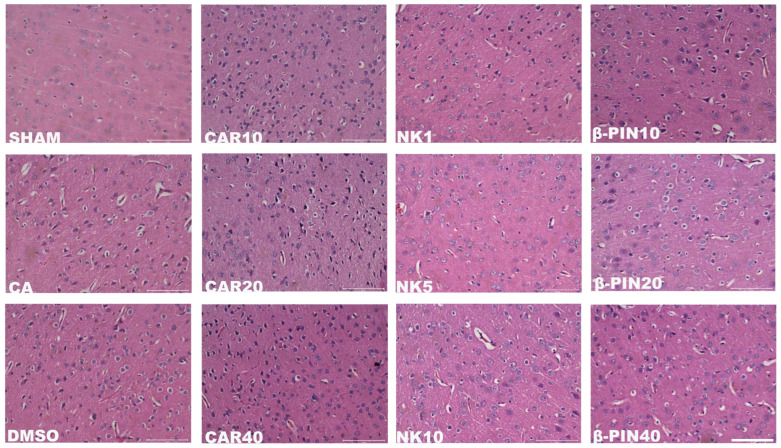
Morphological changes in neurons in the cerebral cortical penumbra revealed by H&E staining in the CA/CPR model (Magnification: ×200; Scale bar: 100 μm).

**Figure 3:**
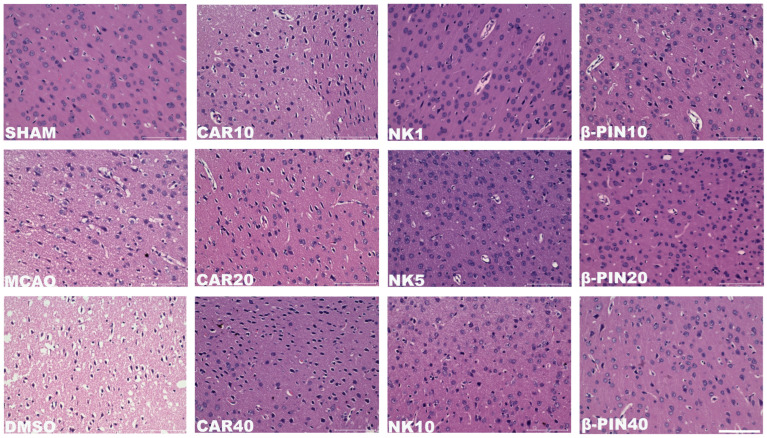
Morphological changes in neurons in the cerebral cortical penumbra revealed by H&E staining in the MCAO model (Magnification: ×200; Scale bar: 100 μm).

As shown in Figure 2, H&E staining of the rat peri-infarct cerebral cortex at 200× magnification revealed a clear and intact peri-infarct cortical structure in the SHAM group. In contrast, the model groups (MCAO and CA/CPR) exhibited disorganized brain tissue in the peri-infarct cerebral cortex, with irregular neuronal cell bodies, vacuolization, and nuclear condensation. These observations confirmed the successful modeling of CIRI. In the treated groups (CAR 10 mg/kg, NK 5 mg/kg, β-PIN 10 mg/kg), these morphological changes in the peri-infarct cerebral cortex were partially alleviated, with the NK 5 mg/kg group showing the most significant improvement. Similar results were obtained in the MCAO model (Figure 3). Therefore, CAR 10 mg/kg, NK 5 mg/kg, and β-PIN 10 mg/kg treatment groups were selected for subsequent experiments.

#### 3.1.2. Nootkatone and β-Pinene May Be the Key Components of PPEO in Antagonizing CIRI

**Figure 4:**
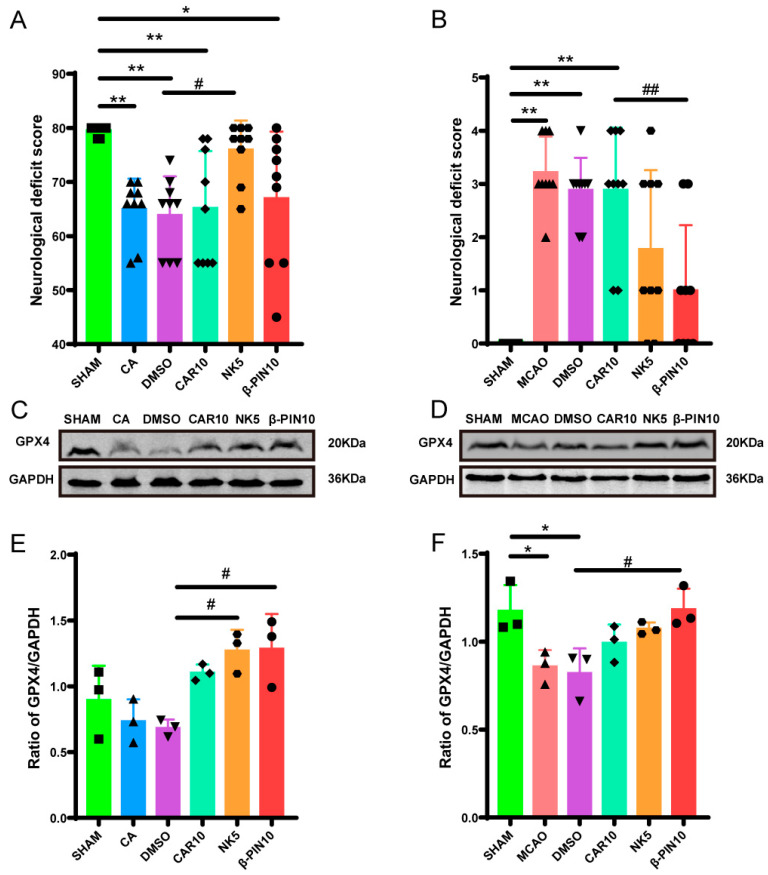
Key component exploration chart. (**A**,**B**) Neurological deficit scores in different groups at 24 h after resuscitation ((**A**) F (5, 48) = 6.345, (**B**) F (5, 48) = 14.95, *n* = 9). (**C**–**F**) Effect of different drugs on expression of GPX4 protein in brain tissue from different groups ((**C**) F (5, 12) = 6.335, (**F**) F (5, 12) = 5.781, *n* = 3) (* and # *p* < 0.05, ** and ## *p* < 0.01).The black squares, circles, and triangles represent independent biological replicate samples within each group.

Neurological function scores were assessed 24 h after model induction to further determine which component of PPEO plays a crucial role in mitigating CIRI. For the two models, different scoring systems were used: the NDS score for the CA/CPR model, where a higher score indicates less neurological damage, and the Zea Longa five-point scale for the MCAO model, where a higher score indicates more severe neurological impairment. In the CA/CPR model, both the CA and DMSO groups had significantly lower neurological function scores compared to the SHAM group (*p* < 0.05), confirming the successful establishment of the model. Compared to the DMSO group, the CAR 10 mg/kg group showed no significant difference, while the NK 5 mg/kg group exhibited a significant increase in score (*p* < 0.05). The β-PIN 10 mg/kg group also showed a trend toward improvement, although the difference was not statistically significant (Figure 4A). In the MCAO model, both the MCAO and DMSO groups had significantly higher neurological function scores than the SHAM group (*p* < 0.05), confirming the successful establishment of the model. Compared to the DMSO group, the CAR 10 mg/kg group showed no significant difference, while the β-PIN 10 mg/kg group showed reduced damage (*p* < 0.05). The NK 5 mg/kg group also showed a trend of improvement, although the difference was not statistically significant (Figure 4B). These results suggest that both Nootkatone and β-pinene possess neuroprotective effects in different degrees of CIRI.

Additionally, the expression of the key ferroptosis protein GPX4 was measured in the brain tissues of the rats 24 h after the surgery. In the MCAO model, GPX4 levels were reduced in both the MCAO and DMSO groups compared to the SHAM group. However, GPX4 levels were significantly elevated in the NK 5 mg/kg and β-PIN 10 mg/kg groups compared to the DMSO group (Figure 4C–F).

These results indicate that the key components of PPEO, likely Nootkatone or β-pinene, exhibit neuroprotective effects. Among them, Nootkatone (NK 5 mg/kg) shows the most pronounced neuroprotective effect, possibly related to the upregulation of GPX4.

### 3.2. Comparative Analysis of Characteristic Changes in Rats with Different Degrees of CIRI Using Metabolomics and Transcriptomics

This study combined serum non-targeted metabolomics with brain tissue transcriptomics to analyze and compare the metabolic changes between focal and global CIRI to gain a deeper understanding of the pathophysiological mechanisms of CIRI and predict the potential targets of neuroprotective drugs. This approach aimed to provide a basis for developing new neuroprotective drugs and precision treatments.

#### 3.2.1. Metabolomics Analysis

**Figure 5:**
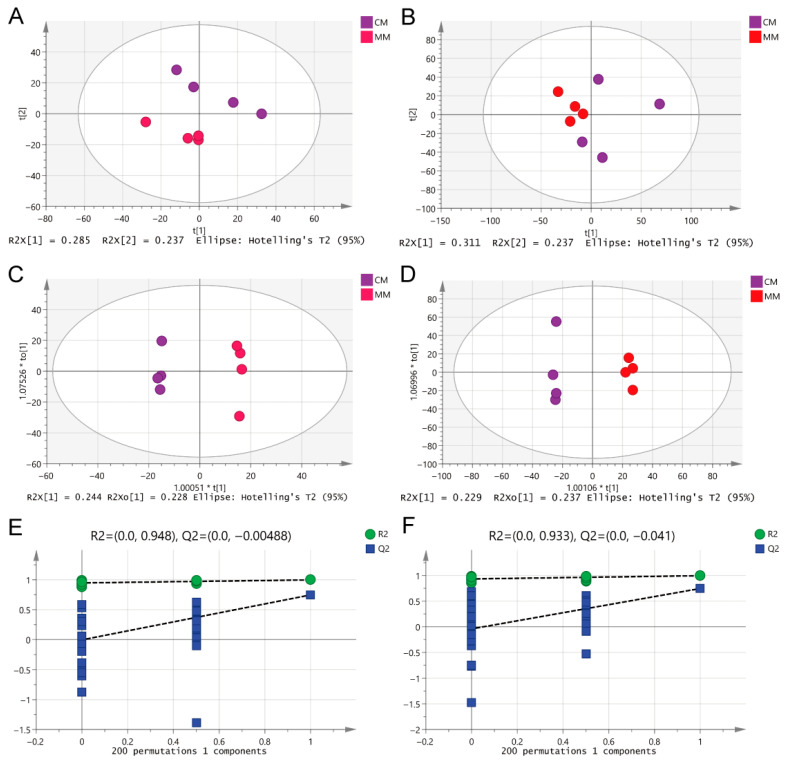
Multivariate statistical analysis of differential metabolite features between MM and CM groups in both ESI modes. (**A**,**B**) PCA of two groups in ESI+ and ESI-modes, respectively. (**C**,**D**) OPLS-DA analysis of the two groups in ESI+ and ESI-modes, respectively. (**E**,**F**) Overfitting test for OPLS-DA model in ESI+ and ESI-modes, respectively.

PCA (Principal Component Analysis) was performed (Figure 5A,B) to first investigate the distribution trends of serum metabolites in rats with different degrees of CIRI. To further differentiate the serum metabolic profiles between the two models, potential biomarkers were selected. An OPLS-DA (Orthogonal Projections to Latent Structures Discriminant Analysis) model was established and analyzed. Compared to PCA, the OPLS-DA score plot more precisely highlighted the metabolic differences between the two groups of rats (Figure 5C,D). After 200 permutation tests, the results confirmed that the model was reliable and not overfitted (Figure 5E,F). The results showed clear clustering within each group, with noticeable inter-group differences. These findings suggest that the serum metabolic profiles of the two models exhibit significant differences in both positive and negative ESI modes.

**Figure 6:**
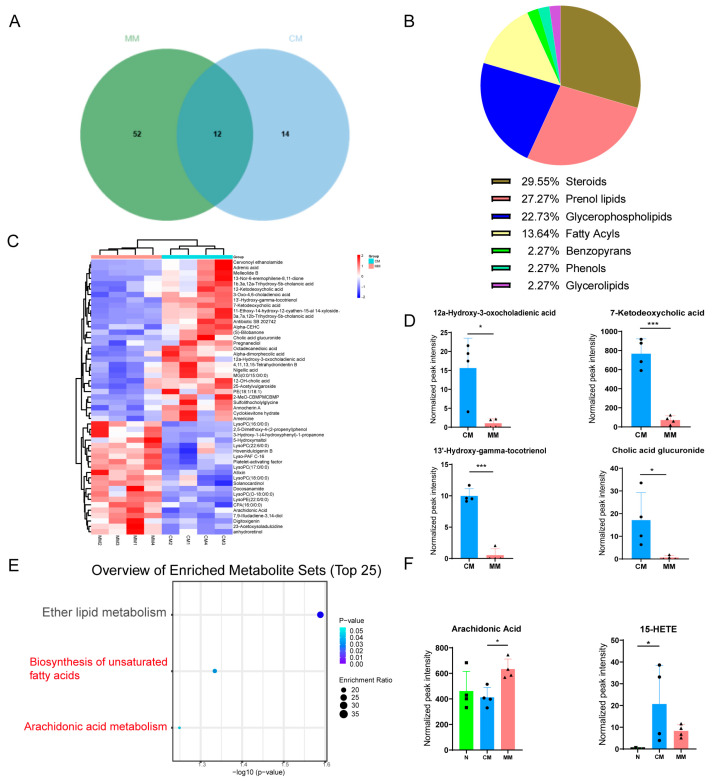
Differential lipid analysis chart. (**A**) The Venn diagram of the differential metabolites between MM and N and the differential metabolites between CM and N. (**B**) The constituent ratio of serum differential metabolite species between CM and MM groups. Steroids (Steroids and steroid derivatives). (**C**) Clustering heatmap analysis of 44 differential metabolite species between CM and MM groups. The color bars represented the log10 value of the ratio for each metabolite species and only statistically significant changes were shown (fold change > 1.50 or <0.67 and *p* < 0.05). (**D**) Trend of metabolites with high differential efficacy between CM and MM Groups. (* *p* < 0.05). (**E**) KEGG pathway enrichment analyses of differential metabolites between CM and MM Groups. (**F**) The normalized peak intensity of Arachidonic Acid, and 15-HETE in each group (F (2, 9) = 4.515, F (2, 9) = 3.983, * *p* < 0.05, *** *p* < 0.001). The black squares, circles, and triangles represent independent biological replicate samples within each group.

Differential metabolites were filtered using a combination of multivariate statistical analysis and the HMDB for endogenous metabolites. The screening criteria were set to score > 42 (out of 60), fold change > 1.50 or <0.67, *p* < 0.05, VIP > 1. Between the MM group and the normal group, 64 endogenous differential metabolites were identified, while 26 differential metabolites were found between the CM group and the normal group (Table S1). Venn diagram analysis showed that 12 of these metabolites were common between the MM and CM groups compared to the normal group, suggesting that these 12 metabolites are related to the common pathophysiological mechanisms of CIRI (Figure 6A). Most of these metabolites were lipids, indicating that lipid metabolism disorder is a common pathological feature of CIRI, which is related to both focal and global CIRI. Further comparison of the MM and CM groups revealed 44 differential metabolites, including 21 metabolites in the ESI-mode and 23 metabolites in the ESI+ mode (Table S2). These metabolites are primarily lipid species, such as steroids (29.55%), prenol lipids (27.27%), glycerophospholipids (22.73%), and fatty acyls (13.64%). A significant proportion of the differential metabolites in the serum from the two models belong to these lipid classes, highlighting lipid metabolism disturbance as a key feature in the pathophysiology of both focal and global CIRI (Figure 6B).

The clustering heatmap analysis of the 44 differential metabolites revealed that each group exhibited good clustering, with relatively small intra-group differences and significant inter-group differences, suggesting the reliability and stability of the metabolites selected (Figure 6C). Comparing the MM and CM groups, lyso-phosphatidylcholine (LPC) was notably more abundant in the MM group serum, further supporting the lipid metabolism alteration as a key feature distinguishing focal from global CIRI. Additionally, we found that certain metabolites, such as 12α-Hydroxy-3-oxocholadienic acid, 7-Ketodeoxycholic acid, 13′-Hydroxy-gamma-tocotrienol, and Cholic acid glucuronide, showed a significant downregulation trend in the MM group (with fold changes >10 and AUC = 1), suggesting that these metabolites can serve as potential biomarkers to differentiate between focal and global CIRI (Figure 6D).

KEGG pathway enrichment analysis was performed on the 44 differential metabolites to investigate the specific metabolic pathways involved with the differential metabolites between the two models. The results showed that the differential metabolites were primarily enriched in three lipid metabolism pathways: the biosynthesis of unsaturated fatty acids (*p* < 0.05), arachidonic acid metabolism (*p* = 0.0572), and ether lipid metabolism (Figure 6E). Among these, arachidonic acid was enriched in both the biosynthesis of unsaturated fatty acids and arachidonic acid metabolism. Furthermore, the levels of the arachidonic acid metabolite 15-HETE were increased in both CIRI models, with higher levels in the CM group serum (*p* < 0.05) (Figure 6F). Interestingly, arachidonic acid plays a crucial role in both the biosynthesis of unsaturated fatty acids and arachidonic acid metabolism, suggesting that arachidonic acid is associated with the metabolic differences between the two models of CIRI.

#### 3.2.2. Transcriptomics Analysis

Transcriptomics analysis was performed on rat brain tissue samples to trace the underlying causes of the differences in lipid metabolism in the two models. A total of 1787 differentially expressed genes (DEGs) were identified, of which 10 DEGs were closely related to the three lipid metabolism pathways enriched in the metabolomics analysis. The results showed that the genes encoding proteins involved in the catabolism of arachidonic acid, such as COX-1 and COX-2 (*PTGS1* and *PTGS2*), were upregulated in the MM group, consistent with the increased levels of arachidonic acid in the MM serum (Table 2). Additionally, the gene expression of phospholipase A2 (*PLA2*) was increased in the MM group, which corresponds to the increased levels of LPC in the MM group. Furthermore, the gene *AGPS*, which is involved in ether lipid synthesis, was also elevated in the MM group.

**Figure 7:**
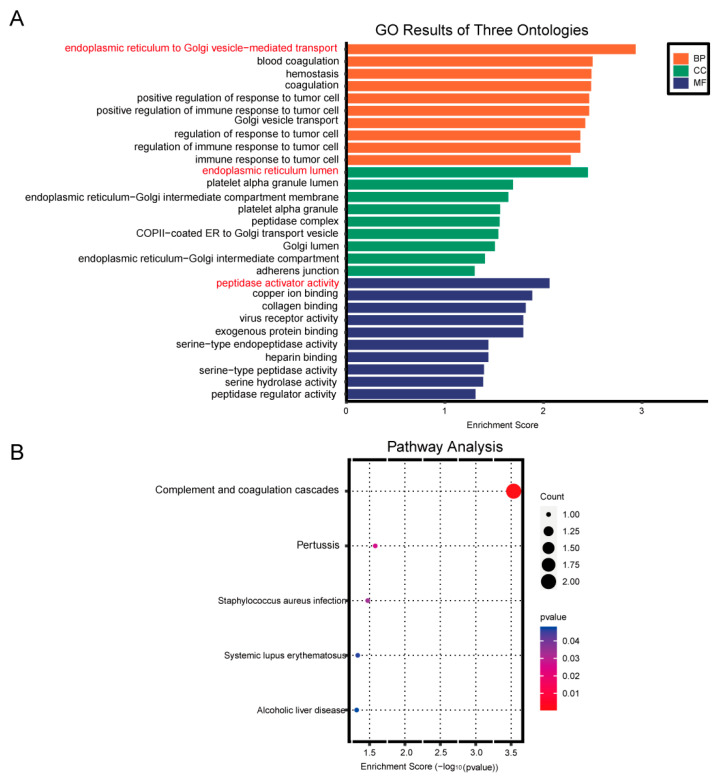
Enrichment analysis chart. (**A**) Gene Ontology (GO) enrichment analysis. (**B**) KEGG pathway enrichment analysis.

Additionally, GO analysis revealed that the biological processes associated with the differentially expressed genes primarily involved the endoplasmic reticulum to Golgi vesicle-mediated transport. The cellular components were predominantly located in the endoplasmic reticulum lumen, while the molecular functions were primarily related to peptidase activity (Figure 7A). KEGG pathway enrichment analysis identified complement and coagulation cascades as the main pathways (Figure 7B).

#### 3.2.3. Animal Experiment Verification of Metabolomics and Transcriptomics Results

**Figure 8:**
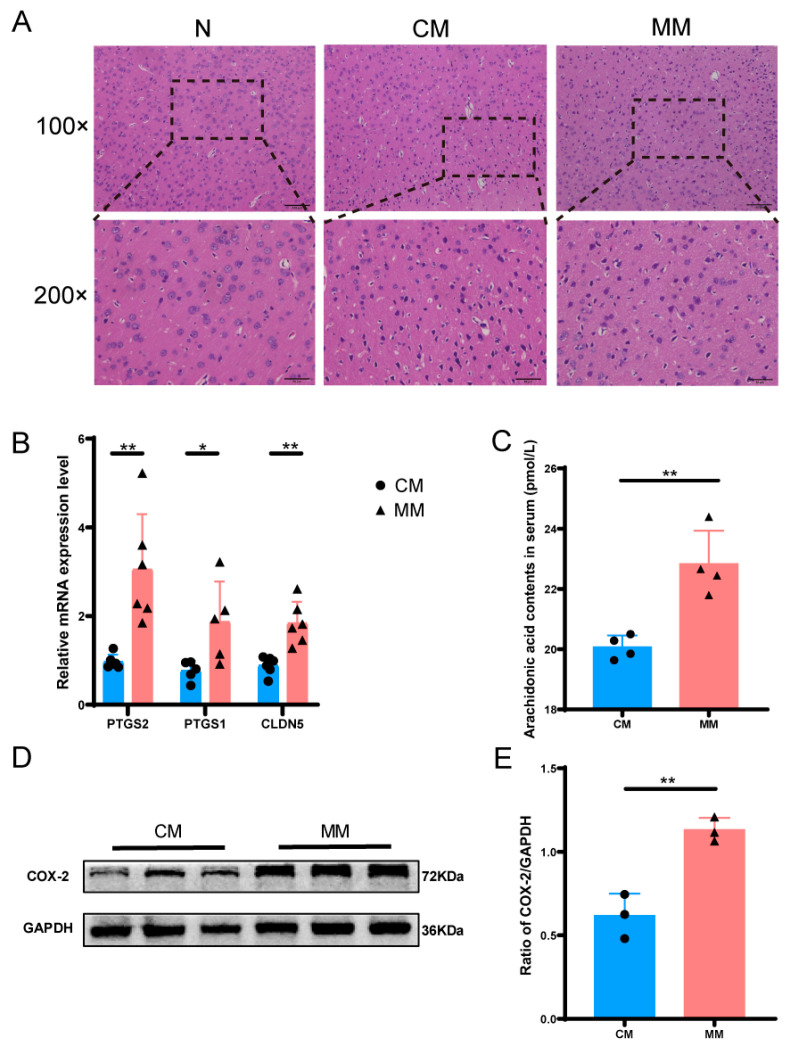
Metabolomics and transcriptomics validation diagrams. (**A**) H&E staining of brain tissue of model rats in each group, 200× was 100× dotted line. (**B**) Relative mRNA expression levels of between CM and MM groups (*n* = 6). (**C**) Arachidonic acid contents in serum between CM and MM groups. (* *p* < 0.05, ** *p* < 0.01) (*n* = 4). (**D**,**E**) Expression of COX-2 protein in brain tissue from different groups (*n* = 3).The black squares, circles, and triangles represent independent biological replicate samples within each group.

The H&E staining results of brain tissue revealed that the N group exhibited a clear and intact cortical structure. In contrast, the CM and MM groups showed disorganized brain tissue with irregular neuronal cell bodies, vacuolization, and increased nuclear condensation, these changes were more pronounced in the MM group, indicating more severe damage compared to the CM group (Figure 8A). To validate the differential expression genes (DEGs) identified in metabolomics and transcriptomics analyses, we performed qPCR to assess genes closely associated with the differential metabolite arachidonic acid. The expression levels of *PTGS1* and *PTGS2* were upregulated in the MM group compared to the CM group. Additionally, the expression of *CLDN5*, a gene related to the blood–brain barrier, also showed an upward trend (Figure 8B), further supporting the transcriptomics results. We measured serum levels of arachidonic acid using ELISA to verify the metabolomics results. The results revealed a significant increase in arachidonic acid levels in the serum of the MM group compared to the CM group, which was consistent with the metabolomics data (Figure 8C). Western blot analysis was performed to examine the protein changes related to arachidonic acid metabolism. The results showed a significant upregulation of COX-2 protein expression in the MM group compared to the CM group (Figure 8D,E).

### 3.3. Nootkatone’s Mechanism of Neuroprotection in the CA/CPR Model

The first two parts of this study have largely identified the potential key components of PPEO responsible for its neuroprotective effects. Lipid metabolism, particularly the disruption of arachidonic acid metabolism, is an important pathophysiological mechanism in cerebral CIRI. Ferroptosis occurs when the ACSL4–LPCAT3–LOXs functional axis is activated, promoting the synthesis and oxidation of polyunsaturated fatty acids (PUFAs) such as arachidonic acid, leading to the explosive accumulation of lipid peroxides, which ultimately results in cell death [22]. This section of the study primarily investigates whether PPEO and its key components can modulate ferroptosis through the regulation of lipid metabolism, thereby exerting neuroprotective effects.

#### 3.3.1. Molecular Docking and CETSA Identifies Nootkatone’s Strong Binding to Ferroptosis-Related Proteins

**Figure 9:**
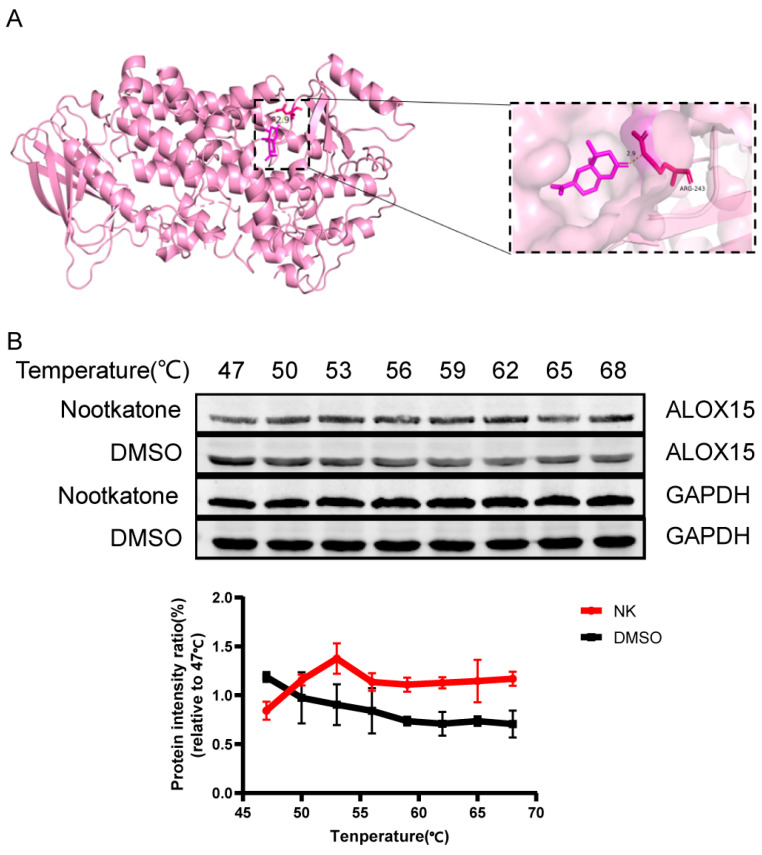
Drug-protein interaction diagram. (**A**) Nootkatone and ALOX15 docking mode diagram. (**B**) Nootkatone and ALOX15 CETSA diagram (*n* = 3).

Molecular docking was performed to evaluate the interaction between Nootkatone and key ferroptosis-related proteins such as GPX4 and ALOX15. The results indicated that Nootkatone had a good binding affinity to both proteins, with binding energies of −5.52 kcal/mol and −7.00 kcal/mol, respectively (Table 3). Furthermore, Nootkatone formed hydrogen bonds with ALOX15, suggesting that it may play a key role in antagonizing ferroptosis (Figure 9A).

CETSA is a technique used to detect the binding efficiency of drugs to target proteins, based on the principle of protein thermal stability [23]. When a drug binds to its target protein, the protein’s structure becomes more stable, making it less prone to denaturation at higher temperatures. Compared to proteins that do not bind to the drug, the drug-bound protein degrades less at the same temperature. In comparison to the DMSO group, the Nootkatone group exhibited higher thermal stability of ALOX15, indicating that Nootkatone binds to ALOX15, which further supports the molecular docking results (Figure 9B).

#### 3.3.2. Comparison of Nootkatone and PPEO’s Neuroprotective Effects

**Figure 10:**
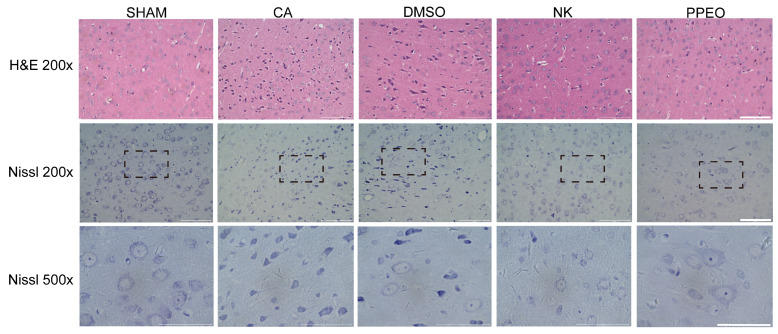
H&E and Nissl staining 24 h after operation, 500× was 200× dotted line. Scale bars: 100 μm (200×), 50 μm (500×).

H&E staining of brain tissues from the CA/CPR model showed that the SHAM group had a well-preserved cortical structure, while the CA and DMSO groups exhibited significant tissue disorganization, irregular neuronal cell bodies, and vacuolization. In the Nootkatone and PPEO treatment groups, these morphological changes were significantly ameliorated (Figure 10). Nissl staining revealed that neuronal density was significantly reduced in the CA and DMSO groups, with some neurons showing dissolution of Nissl bodies (lightly stained cytoplasm and dispersed particles) and shrinkage with hyperchromatic cytoplasm. However, this phenomenon was markedly improved in the Nootkatone and PPEO groups, indicating that both Nootkatone and PPEO exhibited significant neuroprotective effects. Notably, PPEO showed stronger neuroprotective effects compared to Nootkatone (Figure 10).

#### 3.3.3. Nootkatone Reduces Iron, MDA, ACSL4 mRNA Expression Levels in Brain Tissue, and Serum Arachidonic Acid Content After CA/CPR

**Figure 11:**
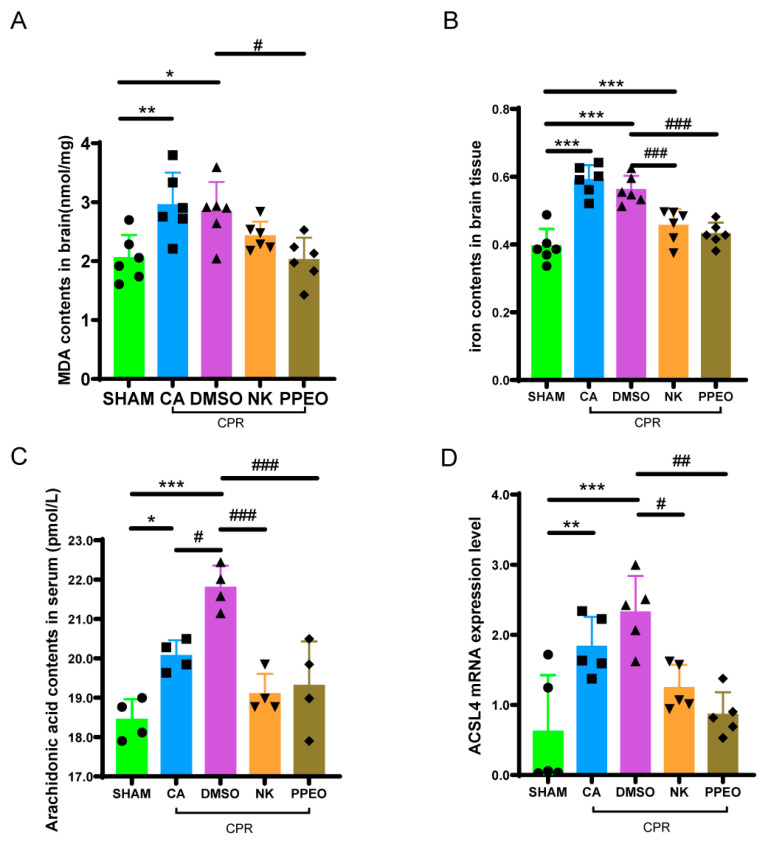
Biochemical index detection and related gene expression. (**A**,**B**) Measurement of level of MDA and total iron in different groups ((**A**) F (4, 25) = 6.159, (**B**) F (4, 25) = 22.21, *n* = 6). (**C**) Arachidonic acid contents in serum (F (4, 15) = 14.59, *n* = 4). (**D**) ACSL4 mRNA expression levels of between different groups (F (4, 20) = 9.404, *n* = 5) (* and # *p* < 0.05, ** and ## *p* < 0.01, *** and ### *p* < 0.001).The black squares, circles, and triangles represent independent biological replicate samples within each group.

Iron and MDA are key biomarkers for detecting ferroptosis, as both levels are elevated during this process. The results showed that both iron and MDA levels were significantly higher in the brain cortical tissue of the CA and DMSO groups compared to the SHAM group. Treatment with Nootkatone and PPEO led to a reduction in both MDA and tissue iron levels, with a more pronounced decrease observed in the PPEO group compared to the Nootkatone group. Additionally, ELISA testing revealed elevated serum levels of arachidonic acid in the CA and DMSO groups, with a statistically significant difference between the groups. Treatment with Nootkatone and PPEO significantly reduced serum arachidonic acid levels (Figure 11A–C). RT-qPCR analysis was performed to measure the relative mRNA expression of ACSL4. The results demonstrated that *ACSL4* expression in brain tissue was significantly increased in the CA and DMSO groups compared to the SHAM group. Treatment with Nootkatone and PPEO significantly reduced the expression of *ACSL4* in brain tissue (Figure 11D).

#### 3.3.4. Nootkatone Reduces LPCAT3 Protein Expression in Brain Tissue After CIRI

**Figure 12:**
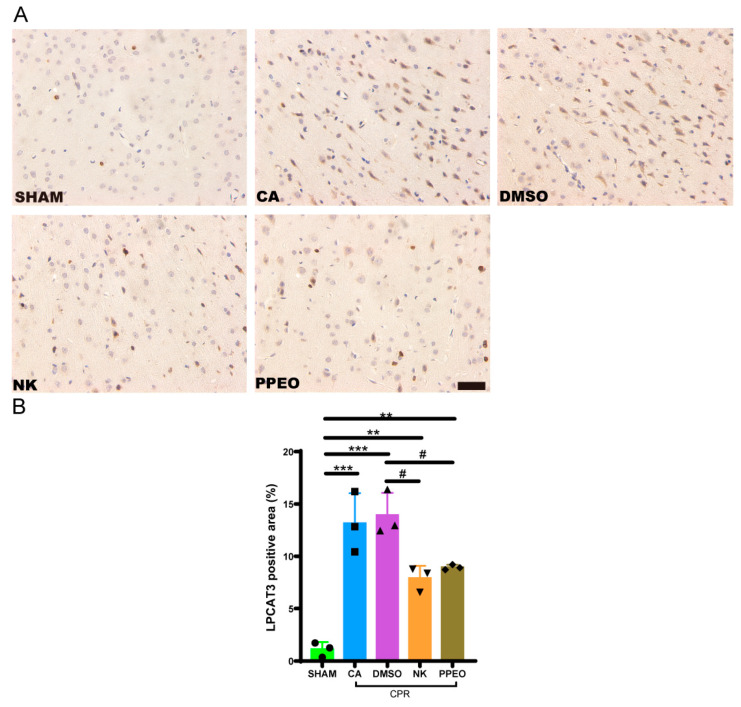
Immunohistochemistry staining (**A**) Immunohistochemistry staining for LPCAT3 in cortex neurons (200× scale is 50 μm) (**B**) Statistical analysis bar graph in each group, respectively (F (4, 10) = 26.40, *n* = 3) (# *p* < 0.05, ** *p* < 0.01, *** *p* < 0.001).The black squares, circles, and triangles represent independent biological replicate samples within each group.

Immunohistochemical analysis of LPCAT3 protein in brain tissue revealed that the positive staining of LPCAT3 was brownish. The expression of LPCAT3 was significantly increased in the CA and DMSO groups, whereas Nootkatone and PPEO treatment resulted in a reduction in LPCAT3 expression. Data analysis showed that both Nootkatone and PPEO reduced the LPCAT3 levels in the brain tissue after CIRI (Figure 12).

#### 3.3.5. Nootkatone Antagonizes Ferroptosis by Regulating GPX4 and the ACSL4–LPCAT3–ALOX15 Axis

**Figure 13:**
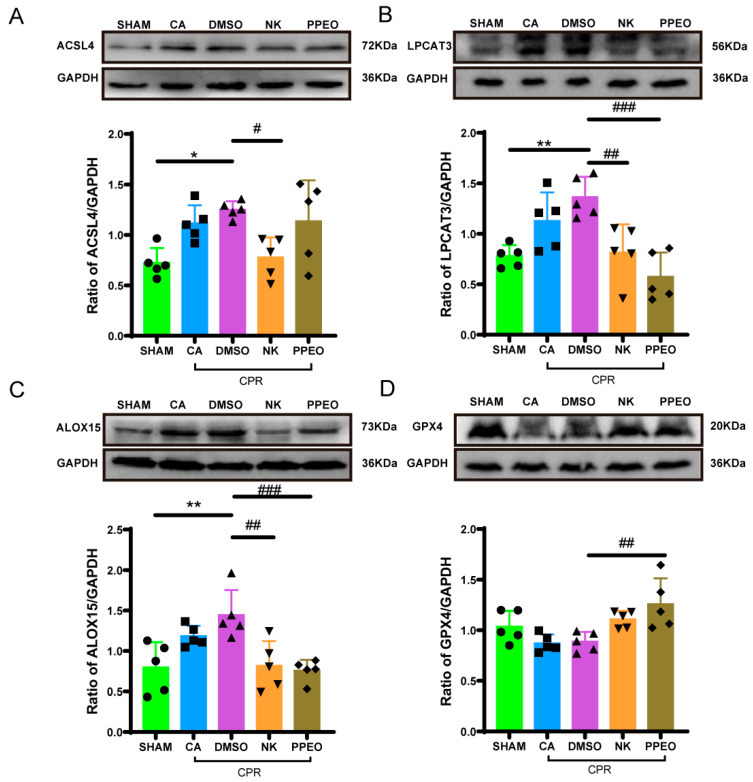
GPX4, LPCAT3, ACSL4, and ALOX15 expression levels of between different groups ((**A**) F (4, 20) = 5.247, (**B**) F (4, 20) = 9.166, (**C**) F (4, 20) = 7.224, (**D**) F (4, 20) = 5.737, *n* = 5) (* and # *p* < 0.05, ** and ## *p* < 0.01, ### *p* < 0.001). The black squares, circles, and triangles represent independent biological replicate samples within each group.

Western blotting results showed that GPX4 expression decreased in the CA and DMSO groups but was restored with Nootkatone and PPEO treatment. In contrast, the protein expression levels of ACSL4, LPCAT3, and ALOX15 were upregulated in the CA and DMSO groups. After treatment with Nootkatone and PPEO, the expression of these proteins decreased, with Nootkatone specifically reducing the levels of ACSL4, LPCAT3, and ALOX15 (except for ACSL4, which remained unchanged in the PPEO group). These results suggest that Nootkatone may inhibit the lipid peroxidation pathway of ferroptosis by regulating the ACSL4-LPCAT3-ALOX15 axis and potentially modulating GPX4 activity (Figure 13).

## 4. Discussion

Cerebral ischemia–reperfusion injury (CIRI) carries high rates of mortality and neurological disability. Although revascularization procedures—such as thrombolysis or thrombectomy—and cardiopulmonary resuscitation are essential to restore blood flow after ischemia, they also trigger pathological cascades that exacerbate brain damage and lead to neuronal death. Ischemic stroke, a form of focal CIRI, ranked as the third leading cause of death globally in 2021 [24]. In cases of global CIRI, such as after cardiac arrest, more than 500,000 people worldwide receive out-of-hospital resuscitation annually. While approximately 22.4% survive to discharge, nearly 20% of survivors experience moderate-to-severe neurological dysfunction [25,26]. Current treatments, including rt-PA and targeted temperature management, remain limited by narrow therapeutic windows and incomplete efficacy [27]. Thus, identifying novel therapeutic targets and developing effective neuroprotective agents against CIRI are urgently needed. This study aims to investigate key mechanisms underlying differential CIRI severity and evaluate potential drug candidates for mitigating neuronal injury.

Guangxi Zhuang Autonomous Region, which abounds in fruits, is renowned for its rich medicinal plant resources, and the concept of “food and medicine homologous” is deeply rooted in local culture. Among the many fruits in the region, Rongxian Shatian pomelo is favored by the public for its sweet flesh. However, its peel remains largely unexplored. Numerous literature reports indicate that the peels of citrus fruits, including Shatian pomelo, are rich in a various bioactive compounds such as terpenes, flavonoids, and phenolic acids, which exhibit anti-inflammatory, antioxidant, and immune-modulatory effects. Similarly, our previous studies demonstrated that PPEO could inhibit ferroptosis induced by erastin and RSL3 in a CA/CPR model via the SLC7A11-GPX4 pathway [8]. PPEO also inhibits TNF-α-induced necroptosis [4], inflammation, and pyroptosis mediated by NLRP3 inflammasome activation [5]. However, which of the PPEO components specifically exerts neuroprotective effects? In the previous study, we used liquid chromatography-mass spectrometry combined with network pharmacology to initially screen three potential key components of PPEO (carvacrol, nootkatone, and β-pinene). Evidence indicates that carvacrol attenuates CIRI through multiple mechanisms, including modulation of the PI3K-Akt signaling pathway [28], inhibition of TRPM7 channel activity [16], and upregulation of GPX4 expression [29]. β-pinene has shown positive effects in neurological diseases, including anxiety [30], depression [31], epilepsy [20], and Alzheimer’s disease [20]. Nootkatone has been shown to alleviate symptoms of Parkinson’s disease through activation of the PI3K/Akt [32] and Nrf2 [33] signaling pathways. Furthermore, it exhibits neuroprotective and anti-inflammatory effects in other neurological disorders, including Alzheimer’s disease [34]. We found that both nootkatone and β-pinene repaired morphological changes in brain tissue and improved neurological function caused by CIRI, in parallel with increasing levels of GPX4. Thus, we initially identified nootkatone and β-pinene as key components to compare the neuroprotective effects in two models, and to further explore the underlying mechanisms.

Disturbed energy metabolism and oxidative stress were core events in the complex pathophysiological cascade of CIRI, but there are limited studies addressing the changes in substance metabolism at different levels of CIRI. It is known to all that the permeability of the blood–brain barrier (BBB) increased during CIRI [35], so metabolites changes in peripheral blood can synchronize with metabolic disturbances in brain tissue. We performed integrated multi-omics analysis on serum and brain tissue samples, respectively, aiming to identify both common features and differences across varying severities of cerebral ischemia–reperfusion injury (CIRI), thereby facilitating a more precise delineation of research priorities. Metabolomics analysis revealed the main differential metabolites were lipids between the two model rats and the normal rats. Several lipids, particularly arachidonic acid (AA), exhibited elevated levels after modeling. It suggests that lipid metabolism disorder is a common pathological feature in CIRI, and the degree correlates with the severity of CIRI. Brain tissue, being rich in lipids, is particularly vulnerable to oxidative stress during the early phase of cerebral ischemia–reperfusion injury (CIRI). Once lipid peroxidation occurs, it inevitably attacks plasma membranes—including mitochondrial membranes—thereby triggering multiple forms of neuronal cell death and resulting in irreversible damage. Therefore, targeting lipid metabolism pathways may be a novel therapy strategy to improve neurological dysfunction after CIRI.

**Figure 14:**
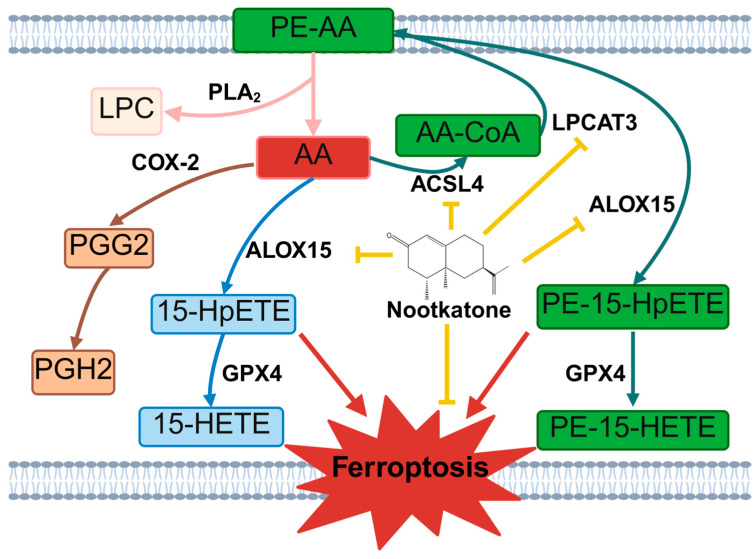
Nootkatone Inhibits Ferroptosis by Regulating Arachidonic Acid Metabolism Pathways. The yellow arrows represent inhibitory effects, the red arrows represent promoting effects, and the other arrows represent substance conversion or enzymatic catalytic directions. PE-AA: Phosphatidylethanolamine-Arachidonic Acid, PLA_2_: Phospholipase A_2_, LPC: Lysophosphatidylcholine, AA: Arachidonic Acid, COX-2: Cyclooxygenase-2, PGG_2_: Prostaglandin G_2_, PGH_2_: Prostaglandin H_2_, ALOX15: 15-Lipoxygenase, 15-HpETE: 15-Hydroperoxyeicosatetraenoic Acid, GPX4: Glutathione Peroxidase 4, 15-HETE: 15-Hydroxyeicosatetraenoic Acid, AA-CoA: Arachidonyl-Coenzyme A, ACSL4: Acyl-CoA Synthetase Long-Chain Family Member 4, LPCAT3: Lysophosphatidylcholine Acyltransferase 3, PE-15-HpETE: Phosphatidylethanolamine-15-Hydroperoxyeicosatetraenoic Acid, PE-15-HETE: Phosphatidylethanolamine-15-Hydroxyeicosatetraenoic Acid.

AA is nutritionally essential for brain function and structure in infants, and exerts regulatory effects on membrane fluidity, signal transduction, and gene transcription. It can only be obtained from the diet and is directly transported to the brain via plasma circulation, and cannot be de novo synthesized. Therefore, the dietary intake of AA is of great importance [36]. As one of the most abundant polyunsaturated fatty acids (PUFAs) in the central nervous system, AA is typically stored within the phospholipid membranes. Under pathological conditions, membrane phospholipids become highly susceptible to oxidative attack. PLA_2_ hydrolyzes the sn-2 ester bond of glycerophospholipids, releasing primarily AA and LPC [37]. AA is primarily metabolized through three major pathways: oxidation by ALOX15 to form the characteristic metabolite 15-HETE, which has been proposed as a biomarker of ferroptosis [38]; catalysis by COX-2 to generate prostaglandins, including prostaglandinE_2_ (PGE_2_) and other inflammatory mediators [39,40]; and reesterification back into membrane phospholipids via the actions of ACSL4 and LPCAT3 (Figure 14). In this study, we observed significant differences in downstream molecules of AA metabolism pathway between the two CIRI models. In the MCAO model, serum levels of AA and LPC were elevated, accompanied by upregulation of PLA_2_ in brain tissue, specific increases in PTGS2 mRNA and COX-2 protein expression, elevated expression of the blood–brain barrier permeability-related gene CLDN5 [41], and more pronounced neuronal damage. In contrast, the CA/CPR model showed a more marked increase in 15-HETE levels. These findings suggest differential engagement of AA metabolic pathways depending on the nature and severity of CIRI: focal CIRI (MCAO) favors COX-2-driven inflammatory responses, whereas global CIRI (CA/CPR) shifts toward ALOX15-mediated ferroptosis. This insight not only provides a rationale for developing precision therapeutic strategies tailored to the type of CIRI, but also strongly provides a sufficient basis for selecting CA/CPR model for subsequent studies on the mechanism of neuroprotective effect of the key components of PPEO.

Both lipid metabolism disorders and ferroptosis are critical pathophysiological mechanisms of CIRI. Ferroptosis is a form of cell death driven by iron-dependent lipid peroxidation, but the exact triggering mechanism is unclear [42]. The ACSL4-LPCAT3-ALOX15 axis is a classical lipid metabolism pathway in ferroptosis [43,44,45]. Some scholars suggested that the accumulation of polyunsaturated fatty acid (PUFA)-derived lipid peroxides directly triggers ferroptosis [46]. Free PUFAs like AA are converted by ACSL4 to AA-CoA which is integrated into membrane phospholipids by LPCAT3, then oxidized by ALOX15, resulting in lipid peroxidation and ferroptosis. Studies have shown that ALOX15 and its product 15-HpETE (an intermediate metabolite formed before ALOX15 catalyzes the conversion of AA to 15-HETE) can exacerbate myocardial ischemia–reperfusion injury by promoting ferroptosis in cardiomyocytes [47]. A clinical study found that ALOX15 is highly expressed in both acute and chronic ischemic hearts of patients [48]. Additionally, isoflavonoids such as genistein can inhibit ALOX15 both in vivo and in vitro, thereby reducing ischemia–reperfusion injury in the heart and cardiomyocytes [49]. Could PPEO and its key components exert neuroprotective effects through inhibiting ferroptosis via ACSL4-LPCAT3-ALOX15 axis?

Molecular docking and CETSA results indicated that Nootkatone has a strong binding affinity with ALOX15, so we compared the neuroprotective effects of Nootkatone and PPEO in the CA/CPR model. Results showed that both of them reduced lipid peroxidation accumulation and iron accumulation in the brain tissue. Furthermore, levels of ACSL4, LPCAT3, and ALOX15 in the model group all increased, and Nootkatone treatment significantly reduced their expression. It suggests that Nootkatone may inhibit ferroptosis by blocking AA incorporation into membrane phospholipids (ACSL4-LPCAT3) and subsequent lipid peroxidation (ALOX15). Also, Nootkatone interrupts the oxidative cascade reaction by upregulating GPX4 activity and reducing lipid peroxides. Additionally, we found that Nootkatone reduces ALOX15 protein levels without affecting mRNA levels. Future studies should investigate whether nootkatone directly facilitates the degradation of ALOX15 or exerts its regulatory effects through upstream signaling pathways, such as Nrf2, NF-κB, or PI3K/Akt.

However, there are several limitations in this study. The sample size in the metabolomics analysis is relatively small, and future research should involve a larger sample size to reduce individual variation. Additionally, the differential metabolites identified through untargeted metabolomics (e.g., arachidonic acid, 15-HETE) have not been validated and quantified using targeted metabolomics, which may compromise the reliability of the metabolomic results. Although we have confirmed the neuroprotective effects of Nootkatone via multiple experimental approaches, further validation in additional preclinical models and clinical trials is still required. Future studies should also focus on exploring whether Nootkatone can synergize with other effective compounds (such as β-pinene) to enhance its neuroprotective efficacy. Furthermore, while Nootkatone has exhibited promising neuroprotective effects in this study, the precise molecular mechanisms underlying its action remain to be fully elucidated. Further investigations are needed to clarify how Nootkatone modulates lipid metabolism, ferroptosis, and oxidative stress to alleviate CIRI.

## 5. Conclusions

In conclusion, this study employed a multi-omics approach to identify dysregulated lipid metabolism as a common pathological mechanism underlying both focal and global CIRI. Furthermore, we revealed that arachidonic acid metabolism may play distinct roles in different forms of CIRI. Importantly, we demonstrated for the first time that Nootkatone, as a key constituent of PPEO, exerts neuroprotective effects by targeting the ACSL4-LPCAT3-ALOX15 axis, thereby suppressing ferroptosis through modulation of lipid metabolic pathways. These findings provide an experimental foundation and candidate compound for the development of lipid metabolism-targeted therapeutic strategies against CIRI (Figure 14).

## Figures and Tables

**Table 1:** Sequences of primers used for RT-qPCR.

Gene	Forward:	Reverse:
*PTGS1*	GGTCTGATGCTCTTCTCCACG	GATGGTTTCCCCTATAAGGATGA
*CLDN5*	GGGCGTCCAGAGTTCAGTTT	ATTCAGCGGTGGTCGTCATC
*PTGS2*	TGGTGCCGGGTCTGATGATG	GCAATGCGGTTCTGATACTG
*ACSL4*	ACCTTCGATCCCAGGAGATT	CTGCTCCAGGGATGTCTATG

**Table 2:** 10 differentially expressed genes between MM and CM groups.

Gene Id	Gene Name	MeanTPM (MM)	MeanTPM (CM)	log2FoldChange	*p* Value	q Value	Result
ENSRNOG00000025121	*Pla2g3*	3.161908	12.579166	−1.99217	0.000614	0.027083	down
ENSRNOG00000012972	*Alox5*	2.468388	6.471839	−1.39061	0.000078	0.007444	down
ENSRNOG00000016945	*Pla2g2a*	0.988476	4.264911	−2.10924	0.009469	0.125761	down
ENSRNOG00000007454	*Aloxe3*	9.318534	3.471569	1.424515	0.005529	0.094329	up
ENSRNOG00000016826	*Pla2g2d*	3.597249	1.177926	1.610646	0.007546	0.112275	up
ENSRNOG00000001701	*Cbr3*	17.132288	8.464055	1.017297	0.015534	0.161850	up
ENSRNOG00000002525	*Ptgs2*	45.577018	12.498085	1.866599	0.001528	0.045633	up
ENSRNOG00000007415	*Ptgs1*	15.080736	3.300183	2.192089	0.000034	0.004082	up
ENSRNOG00000001547	*Agps*	19.082327	7.161477	1.413908	0.004368	0.082598	up
ENSRNOG00000043192	*Hacd1*	4.286617	1.713449	1.322936	0.045832	0.274355	up

**Table 3:** PPEO active ingredients and core target docking score table (kcal/mol).

	CAR	NK	β-PIN
GPX4	−4.44	−5.52	−4.49
ALOX15	−5.90	−7.00	−5.50

## Data Availability

The original contributions presented in this study are included in the article/Supplementary Material. Further inquiries can be directed to the corresponding authors.

## Supplementary Materials

The following supporting information can be downloaded at https://www.mdpi.com/article/10.3390/cimb47110912/s1.
