# Supplementary material for: Key Components of PPEO in Antagonizing Cerebral Ischemic Reperfusion Injury in Rats by Regulating Ferroptosis Through Arachidonic Acid Metabolic Pathway
Source: Curr Issues Mol Biol. 2025 Nov 3;47(11):912. doi: 10.3390/cimb47110912 (PMC12651068; doi:10.3390/cimb47110912)
Supplement: Supplementary file 1 [file cimb-47-00912-s001.zip › Supplementary Materials1.pdf]

Table S1 Table of Venn diagrams regarding differential metabolites.

MM	CM	Intersection
PC(18:0/22:6)	LysoPC(O-18:0/0:0)	LysoPC(14:0/0:0)
LysoPC(22:6/0:0)	LysoPC(22:1/0:0)	Glyceryl 5-hydroxydecanoate
1-O-Hexadecyl-sn-glycero-3-phosphocholine	LysoPE(22:0/0:0)	3-Indolepropionic acid
Stearoylcarnitine	23-Acetoxysoladulcidine	LysoPC(16:0/0:0)
SM(d18:1/16:0)	1-Hexadecanol	LysoPC(16:1/0:0)
Behenoylglycine	Solanocardinol	(3beta,5beta,8beta,22E,24xi)-Ergosta-6,22-diene-3,5,8-triol
Adrenic acid	Ganoderic acid Mk	PC(14:0/0:0)
12-Ketodeoxycholic acid	CPA(16:0/0:0)	LysoPE(0:0/18:0)
Cervonoyl ethanolamide	LysoPC(18:1/0:0)	Tetrahydrocortisone
Nutriacholic acid	3b,15b,17a-Trihydroxy-pregnenone	Alpha-CEHC
Hovenidulcigenin B	LysoPC(18:0/0:0)	Sphinganine 1-phosphate
LysoPE(0:0/16:0)	Melleolide B	Porrigenin A
LysoPC(20:4/0:0)	Annuolide G	
Oleoylcarnitine	LysoPE(18:1/0:0)	
LysoSM(d18:1)		
Alpha-dimorphecolic acid		
11-Oxohexadecanoic acid		
Octadecanedioic acid		
2'-Apo-beta-carotenal		
Sphingosine 1-phosphate		
MG(0:0/15:0/0:0)		
1-Stearoylglycerophosphoserine		
Cholestane-3,7,12,25-tetrol-3-glucuronide		
14,20-Epoxy-17-hydroxy-1-oxowitha-3,5,24-trienolide		
PE(DiMe(13,5)/DiMe(11,3))		
12-Hydroxyoctadecanoic acid		
(Z)-8-Tetradecenal		
Sterol		
20a-Dihydroprogesterone		
Docosahexaenoic acid		
(ent-2alpha,3beta,15beta,16beta)-15,16-Epoxy-2,3-kauranediol		
Cholic acid glucuronide		
15(S)-HETE		
Annocherin A		
17-Hydroxypregnenolone sulfate		

MM	CM	Intersection
3a,7a,12b-Trihydroxy-5b-cholanoic acid		
11-Ethoxy-3,4-epoxy-14-hydroxy-12-cyathen-15-al 14-xyloside		
p-Cresol sulfate		
(3beta,22E,24R)-3-Hydroxyergosta-5,8,22-trien-7-one		
Ganodermic acid P2		
Palmitic acid		
PE(18:1/18:1)		
9,10-DHOME		
16-Hydroxy-10-oxohexadecanoic acid		
PE(18:0/18:3)		
12-Ketoporrigenin		
Pangamic acid		
Copalic acid		
4,11,13,15-Tetrahydroridentin B		
1alpha,23(S),25-trihydroxyvitamin D3		
2-(Methoxycarbonyl)-5-methyl-2,4-bis(3-methyl-2-butenyl)-6-(2-methyl-1-oxopropyl)-5-(4-methyl-3-pentenyl)cyclohexanone		
8-Heptadecenal		

Table S2 44 differential metabolite species between MM and CM groups in both ESI modes.

Metabolite species	Formula	Scan mode	Detective *m/z*	RT (min)	*P* value	Fold change
anhydroretinol	C20H28	ESI +	269.22611	14.65	0.019	2.52
LysoPE(22:0/0:0)	C27H56NO7P	ESI +	538.38598	14.41	0.000	2.27
LysoPC(O-18:0/0:0)	C26H56NO6P	ESI +	510.39089	14.14	0.003	2.14
CPA(16:0/0:0)	C19H37O6P	ESI -	391.22569	23.16	0.011	2.08
LysoPC(16:0/0:0)	C24H50NO7P	ESI +	496.33816	11.95	0.005	1.99
7,9-Illudadiene-3,14-diol	C15H22O2	ESI +	235.16925	14.60	0.000	1.77
Docosanamide	C22H45NO	ESI +	340.35700	16.99	0.034	1.75
2,5-Dimethoxy-4-(2-propenyl)phenol	C11H14O3	ESI +	195.10167	10.94	0.032	1.71
LysoPC(18:0/0:0)	C26H54NO7P	ESI -	522.35572	14.38	0.012	1.68
Lyso-PAF C-16	C24H52NO6P	ESI +	482.35968	12.90	0.007	1.67
Solanocardinol	C27H45NO3	ESI +	454.32873	11.67	0.014	1.66
Digitoxigenin	C41H64O17	ESI +	851.39743	10.90	0.032	1.60
23-Acetoxysoladulcidine	C29H47NO4	ESI +	496.33932	14.19	0.019	1.55
Arachidonic Acid	C20H32O2	ESI +	305.24694	14.66	0.007	1.53
LysoPC(22:6/0:0)	C30H50NO7P	ESI +	568.33914	12.05	0.030	1.53
Hovenidulcigenin B	C32H50O7	ESI +	569.34250	11.85	0.038	1.52
LysoPC(17:0/0:0)	C25H52NO7P	ESI +	510.35474	12.96	0.000	1.51
Platelet-activating factor	C26H54NO7P	ESI +	524.37022	13.58	0.003	1.51
25-Acetylvulgaroside	C27H42O7	ESI -	459.27167	10.00	0.040	0.49
Nigellic acid	C15H20O5	ESI -	261.11229	10.43	0.030	0.47
Pregnanediol	C21H36O2	ESI -	319.26321	15.57	0.040	0.41
Alpha-CEHC	C16H22O4	ESI -	313.11877	8.64	0.021	0.40
4,11,13,15-Tetrahydroridentin B	C15H24O4	ESI -	249.14882	10.85	0.020	0.38
12-OH-cholic acid	C24H38O4	ESI -	389.26903	10.17	0.035	0.37
Annocherin A	C24H38O5	ESI -	405.26391	9.65	0.005	0.35
Sulfolithocholylglycine	C26H43NO7S	ESI -	512.26812	7.98	0.038	0.33
Alpha-dimorphecolic acid	C18H32O3	ESI -	295.22713	12.80	0.032	0.31
1b,3a,12a-Trihydroxy-5b-cholanoic acid	C24H40O5	ESI +	431.27699	9.97	0.031	0.29
12-Ketodeoxycholic acid	C24H38O4	ESI +	391.28446	9.95	0.037	0.28
MG(0:0/15:0/0:0)	C18H36O4	ESI -	297.24243	12.47	0.005	0.27
Octadecanedioic acid	C18H34O4	ESI -	295.22666	12.38	0.002	0.26
3-Oxo-4,6-choladienoic acid	C24H34O3	ESI +	371.25792	9.88	0.009	0.26
PE(18:1/18:1)	C41H78NO8P	ESI -	788.54255	11.65	0.025	0.24
3a,7a,12b-Trihydroxy-5b-cholanoic acid	C24H40O5	ESI -	453.28489	9.07	0.002	0.20
Melleolide B	C24H32O7	ESI -	431.20995	7.10	0.015	0.17
11-Ethoxy-14-hydroxy-12-cyathen-15-al 14-xyloside	C27H42O8	ESI -	475.26692	9.07	0.002	0.15
2-MeO-CBMPMCBMP	C29H46O4	ESI -	457.33148	11.15	0.044	0.14
13-Nor-6-eremophilene-8,11-dione	C14H20O2	ESI +	221.15372	10.08	0.030	0.12
Cervonoyl ethanolamide	C24H36O3	ESI +	373.27340	9.18	0.045	0.11

**Metabolite species**	**Formula**	**Scan mode**	**Detective *m/z***	**RT (min)**	***P* value**	**Fold change**
Adrenic acid	C22H36O2	ESI +	355.26280	9.18	0.047	0.11
7-Ketodeoxycholic acid	C24H38O5	ESI -	405.26388	9.21	0.000	0.09
12a-Hydroxy-3-oxocholadienic acid	C24H34O4	ESI +	387.25285	9.70	0.011	0.07
13'-Hydroxy-gamma-tocotrienol	C28H42O3	ESI -	471.31024	11.37	0.000	0.05
Cholic acid glucuronide	C30H48O11	ESI -	583.31028	8.06	0.034	0.04
