# Supplementary material for: Key Components of PPEO in Antagonizing Cerebral Ischemic Reperfusion Injury in Rats by Regulating Ferroptosis Through Arachidonic Acid Metabolic Pathway
Source: Curr Issues Mol Biol. 2025 Nov 3;47(11):912. doi: 10.3390/cimb47110912 (PMC12651068; doi:10.3390/cimb47110912)

Unedited Western Blot (WB) Images.

1.Effect of different drugs on expression of GPX4 protein in brain tissue from different groups.

CA/CPR model

SHAM CA DMSO CAR10 NK5 β-PIN10

CA/CPR

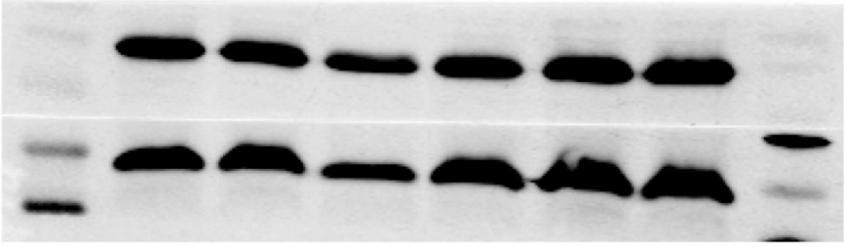

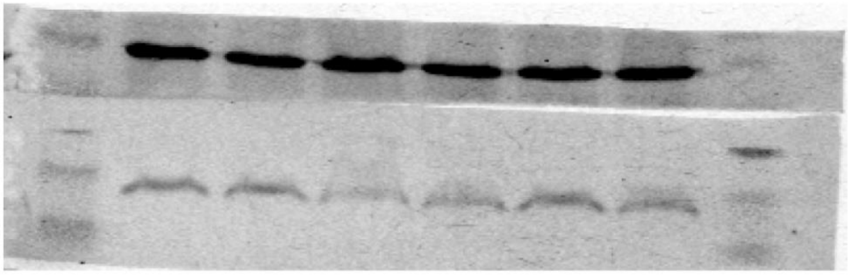

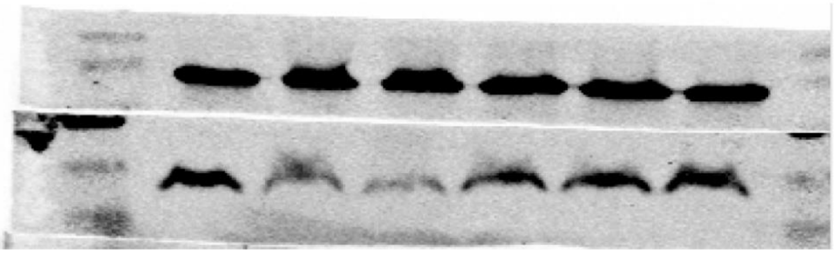

MCAO model

SHAM MCAO DMSO CAR10 NK5 β-PIN10

MCAO

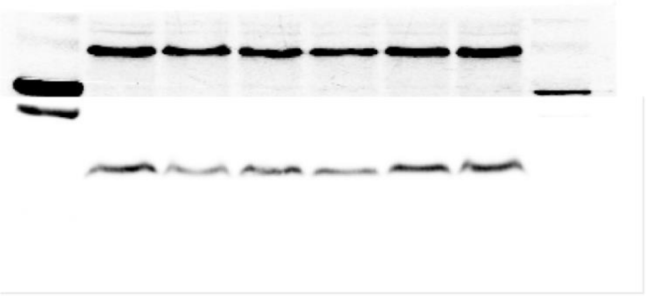

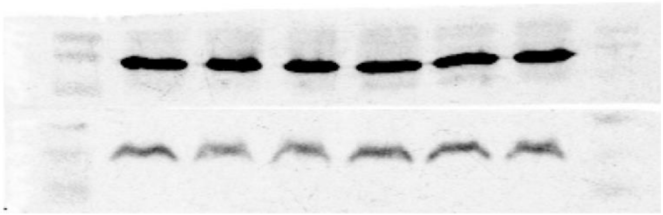

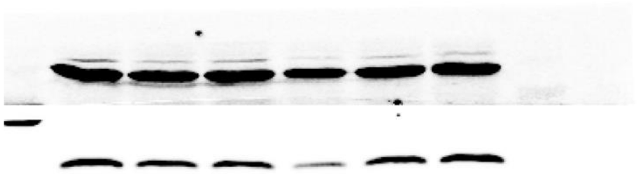

2.Expression of COX-2 protein in brain tissue from different groups.

CM1 CM2 CM3 MM1 MM2 MM3

COX-2

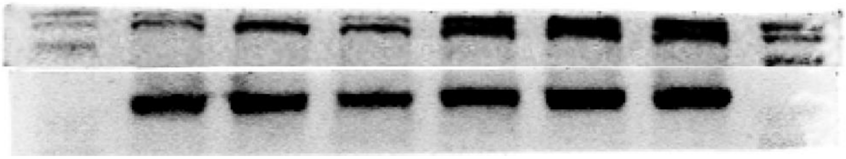

3.Nootkatone and ALOX15 CETSA diagram

Nootkatone group

47 50 53 56 59 62 65 68°C

Nootkatone group

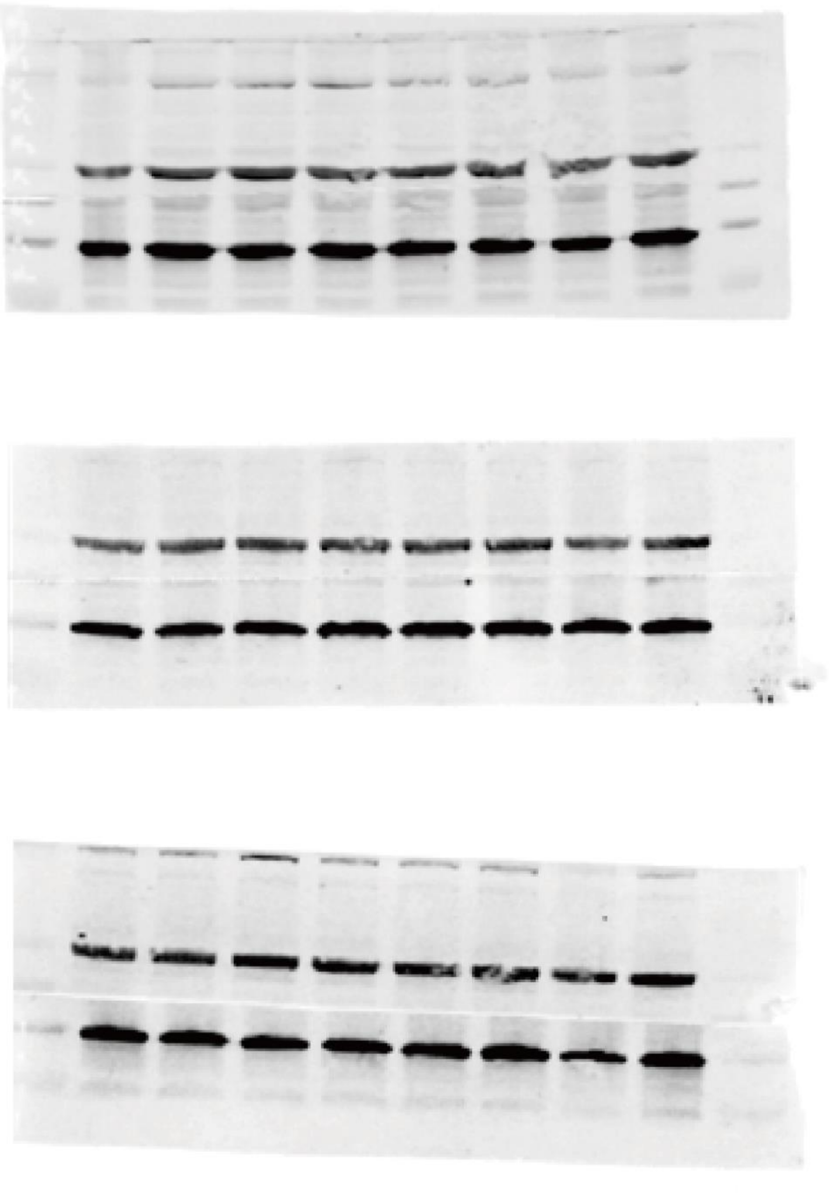

DMSO group

DMSO group

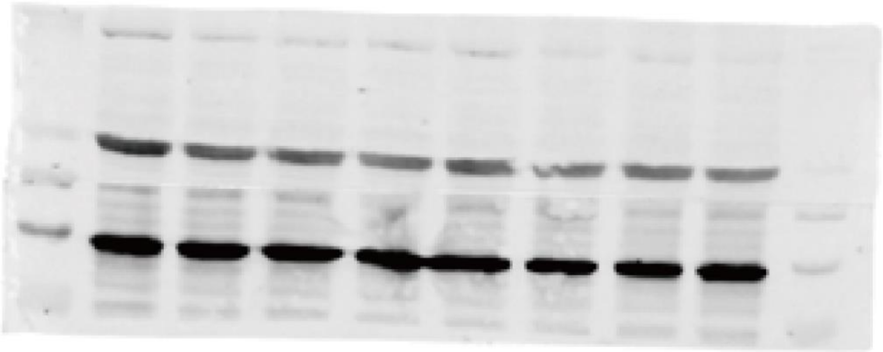

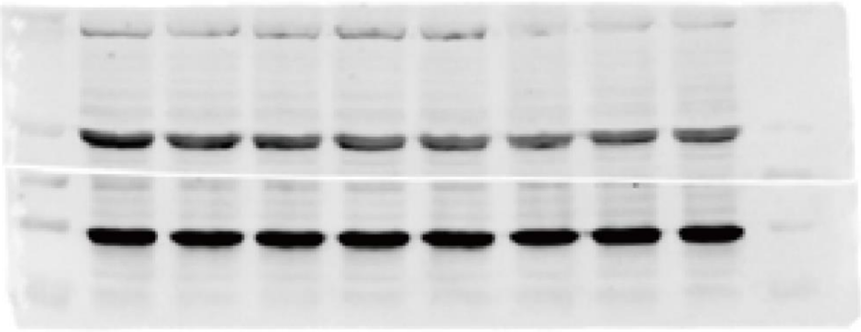

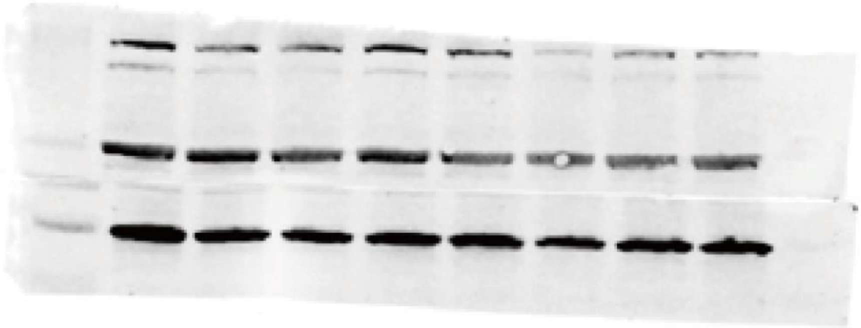

4.GPX4, LPCAT3, ACSL4, and ALOX15 expression levels of between different groups

GPX4

SHAM CA DMSO NK PPEO

GPX4

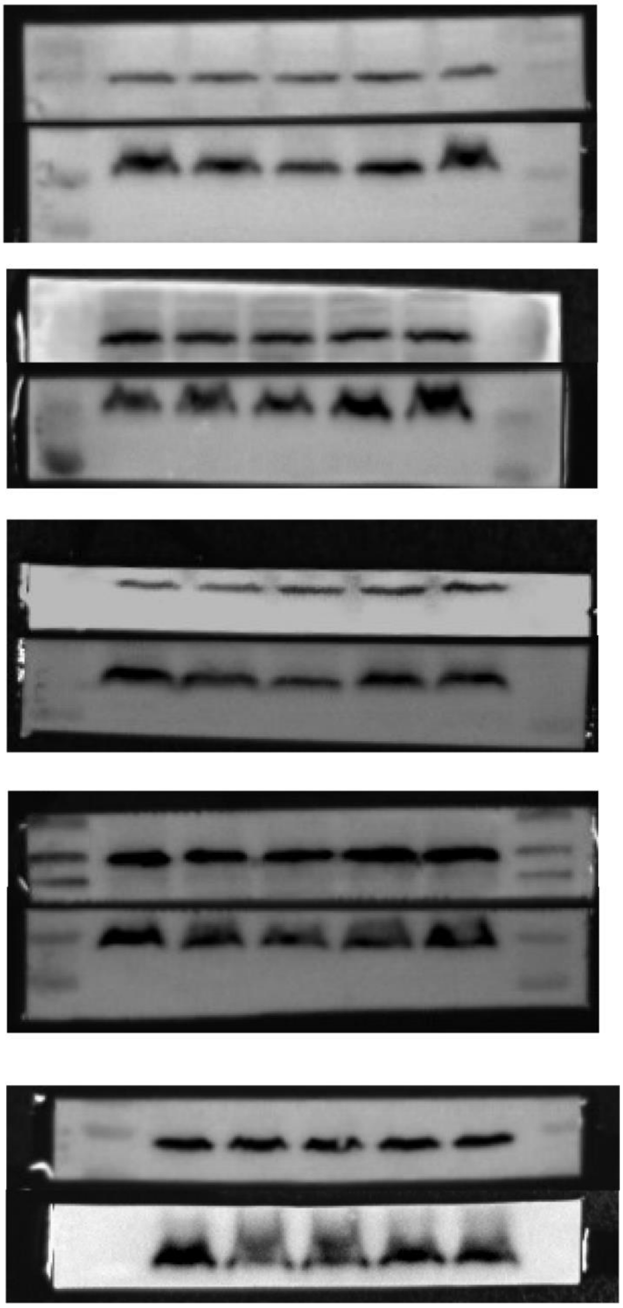

LPCAT3

LPCAT3

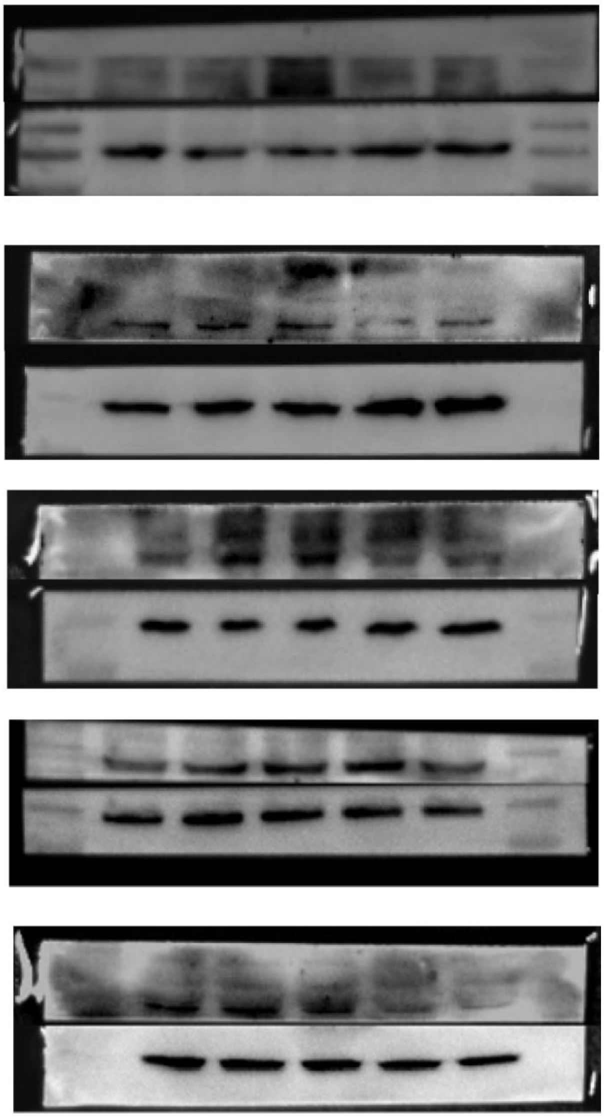

ALOX15

ALOX15

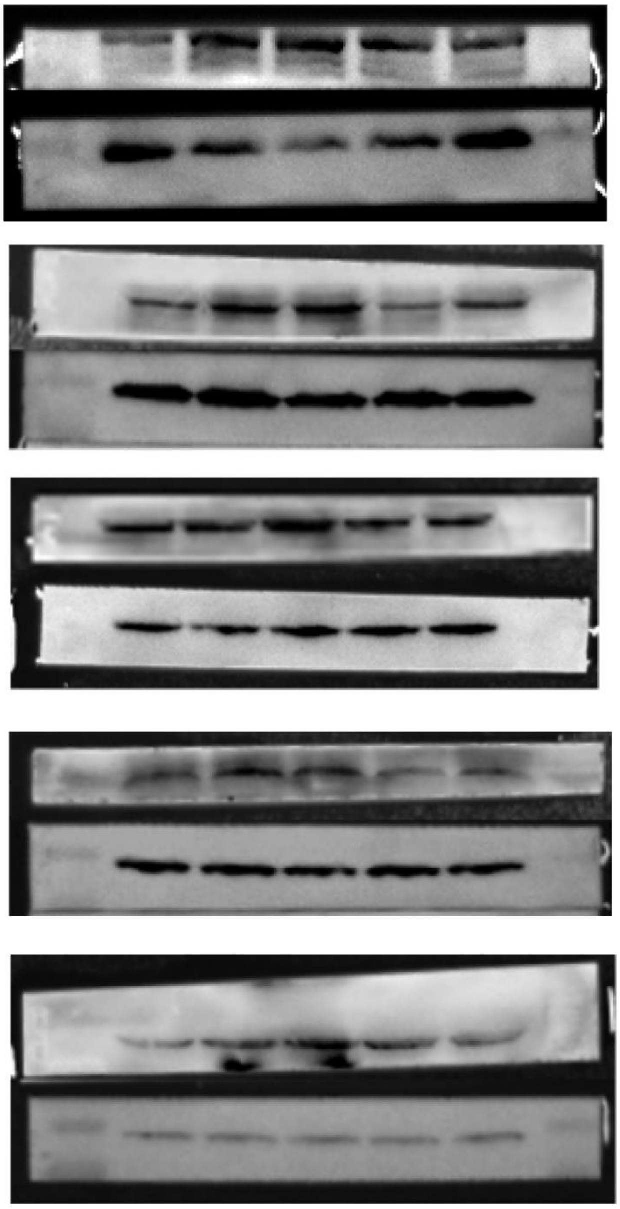

ACSL4

ACSL4

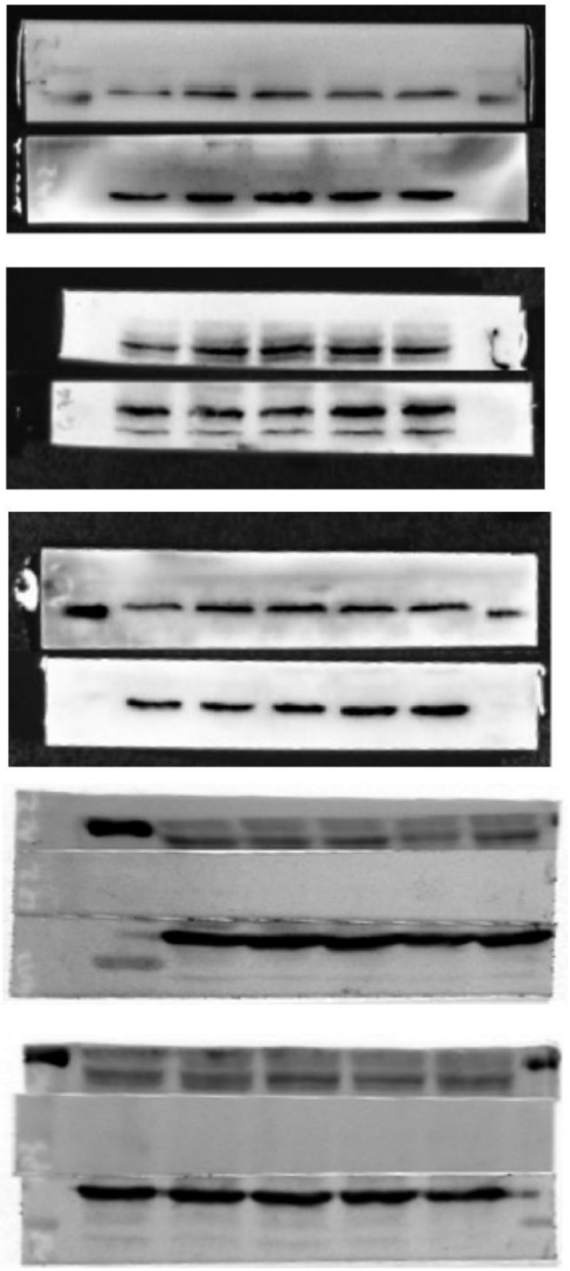

Supplement: Supplementary file 1 [file cimb-47-00912-s001.zip › Supplementary Materials2.pdf]
